# Concept mapping sociocultural aspects of cervical cancer prevention among African American women

**DOI:** 10.3389/fpubh.2023.1311286

**Published:** 2024-02-02

**Authors:** Chakema Carmack, Angelica Roncancio, Taylor M. Coleman, Sarah McKay

**Affiliations:** ^1^HEALTH Research Institute—RCMI, University of Houston, Houston, TX, United States; ^2^Psychological Health and Learning Sciences Department, College of Education, University of Houston, Houston, TX, United States; ^3^Health and Behavioral Sciences, Department of Social Sciences, University of Houston—Downtown, Houston, TX, United States

**Keywords:** cervical cancer, HPV, African American, health promotion, health messaging

## Abstract

**Introduction:**

For African American women in Houston, cervical cancer mortality is disproportionate to their racial and ethnic counterparts. Most notably, lack of human papillomavirus (HPV) screening and vaccination as well as late diagnosis increase cervical cancer mortality. However, cervical cancer is largely preventable. While previous research has identified a few social determinants that are specifically related to cervical cancer (e.g., education, income, neighborhood), there may be a host of additional social and cultural factors that contribute to a lack of preventative behavior.

**Methods:**

The present study used concept mapping to explore sociocultural determinants of cervical cancer prevention beliefs among young African American women. *N* = 15 African American women, ages 18–25, participated in a group concept mapping session focused on cervical cancer knowledge, beliefs about women’s health, and how their social environment and culture play a role in their conceptualization of cervical cancer prevention.

**Discussion:**

Five overarching concepts emerged: (1) Screening, (2) Support System, (3) Cervical Cancer 101 (knowledge), (4) Fatalism, and (5) Ease of Prevention.

**Conclusion:**

The present study highlights the use of concept mapping for prevention science, particularly in exploratory studies for understanding cervical cancer screening barriers, avenues for intervention, and public health messaging. We discuss the findings and implications for public health research in cervical cancer prevention tailored for African American women.

## Introduction

In the U.S., African American women (AAW) have the highest rates of cervical cancer mortality relative to their racial counterparts (1). AAW in Harris County, Houston, Texas are also at higher risk of cervical cancer mortality. Currently, the age-adjusted cervical cancer mortality rate among AAW in Harris County is 3.7/100,000, which is higher than the rate of Houston and Texas of 2.9/100,000 each. In Houston, the rate of cervical cancer mortality among AAW is the highest relative to their White counterparts with a rate of 2.4/100,000, which is 30% lower (2). In 2020, Houston had a 24% African American population, higher than the national average of 12% (3). Houston is an excellent location to engage in research focused on achieving cervical cancer equity and impacting prevention among AAW. The consequences of cervical cancer mortality in this population go far beyond the individual affected. It affects the entire family structure and, at its core, the entire community.

According to the U.S. Preventive Services Task Force (USPSTF), two screening tests are used to identify pre-cancerous cells or cervical cancer at an early stage: the Pap test and the human papillomavirus (HPV) test. The Pap test is a cytology test that checks for cervix cell abnormalities, while the HPV test is a laboratory blood test that checks for DNA and RNA of certain strains of HPV. Currently, the U.S. Preventive Services Task Force (USPSTF) recommends cervical cancer screening for women ages 21 to 65 years old. Cervical cancer screening guidelines are age group specific for females of average risk. It is recommended that women ages 21–29 should have a Pap test alone every 3 years. Females, ages 30–65 have three recommended options for cervical cancer screening: Pap test alone every 3 years; HPV test alone every 5 years; or combination HPV and Pap test every 5 years (4).

Cervical cancer is highly preventable (4). However, it was found that African American Houstonian women were 45–53% less likely than Hispanic women to schedule a follow-up appointment following a low-grade abnormal pap screening (5), which is an efficacious test for detecting pre-cancerous cells. Factors related to cervical cancer risk include lack of cervical cancer screening (e.g., Pap test) and low uptake of the HPV vaccine, among others (6–8). Although the HPV vaccine has been available since 2006 and recommended for female girls ages 15–26 (2), uptake among AAW remains low (6). Knowledge, education, and other social determinants of health, such as public health awareness and access to care are also key factors influencing risk for AAW (9–13). These individual-level and social determining factors are important to consider. However, other social and cultural factors may have strong interconnections with prevention behavior and are in need of further investigation (14). In addition to the social determinants of health, socioculture is important as well. For instance, issues surrounding mistrust of medical systems (15–17) and perspectives about fatalism (18, 19) may be culture-bound and affect health behavior. It is likely that qualitative inquiry will yield other sociocultural determinants.

The present study utilized concept mapping in determining salient sociocultural determinants of cervical cancer prevention beliefs among AAW. Concept mapping is a mixed-methods, systems-thinking methodology that consists of a card-sorting activity that will identify and “map out” salient sociocultural factors of prevention, as conceptualized by the participants themselves. It has been used widely in nursing research (20–22), to understand screening barriers from multiple stakeholder perspectives such as academics, medical professionals, and patients (23, 24), and with diverse populations or hard-to-reach populations (25, 26). Concept mapping will enable us to visually observe how AAW perceive the importance, barriers, and sociocultural influences of cervical cancer prevention, as well as their relation to one another.

## Methods

### Participants

The sample included *N* = 15 African American women. Participants’ ages ranged from 18 to 25 years old; the mean age was 22 years old. The majority of the participants (93%; *N* = 14) reported being single at the time of participation and N = 1 reported being married; 80% (*N* = 12) reported being heterosexual and 13% (*N* = 2) reported being bi-sexual. The majority of the participants reported some college, with 67% (*N* = 10) reporting that they were currently in college, 20% (*N* = 3) reporting a college degree, and 13% (*N* = 2) reporting no college. Regarding access to care, 47% (*N* = 7) reported having independent private insurance, 20% (*N* = 3) reported having private insurance via their parents/guardians, and 33% (*N* = 5) reported having no health insurance. No participant reported having a single dose of the HPV vaccination series; while 47% (*N* = 7) reported having had a Pap test in the past, and 53% (*N* = 8) reported never having a Pap test.

### Procedure

Study eligibility included: self-identification as biological female, self-identification as African American, over age 18 and, able to speak/read English. Exclusion criteria included a history of cervix dysplasia. Participants were recruited through postings on community bulletin boards within African American communities, at events held by the university’s HEALTH Research Institute Research Center in Minority Institutions (HRI-RCMI), and on HRI-RCMI social media sites. All study procedures took place at the University campus in one visit in May 2022. Participants gathered in a closed-door conference room on a Saturday afternoon for the concept mapping procedure. Due to the anticipation of COVID-variant precautions at the time, the room was large enough to implement social distancing protocols and face masks were encouraged for campus visits and interactions. Participants were informed and consented via written consent to take the brief anonymous demographic survey.

The concept mapping procedure consists of several phases. In phase 1, the information generating phase, the participants collectively generated statements based on the following general prompts: *What are some community values that encouraged caring about your possible risk of cervical cancer? What are some things that would make it easier to stay up to date with your Pap test? What are some things you wish you were taught about cervical cancer prevention and women’s health?* Participants were given a short-list of relevant definitions to ensure health literacy (e.g., Cervix, cervical cancer, HPV, Pap test, infection, culture, significant others, media, clinic/doctor’s office). Responses were listed in real-time using a computerized whiteboard projector. The group setting allowed participants to reciprocate responses and generate ideas from one another. This phase took approximately 40 min. Participants took a 30-min intermission break in order for researchers to gather the statements that were developed and print the generated phrases on cards and rating sheets in preparation for phases 2 and 3. Table 1 shows the card statements used for the card sorting and rating phases (phases 2 and 3).

**Table 1:** Overarching concepts and individual concepts—identification and ratings.

Concept	Card #	Rating		Card #	Rating
Screening		3.64	Support system		3.59
I am familiar with the Pap test	16	3.6	Health insurance would make cervical cancer screening easier	68	4.1
Screening is important only if cancer runs in your family	17^±^	4.2^a^	My mother taught me about women’s health	3	2.5
Cervical cancer is an issue for Black women, in particular	8	3.2	I have someone “safe” to talk to about women’s health issues	12^±^	3.8
Cervical cancer was discussed in sex ed./PE/health class	67	2.4	There are commercials about cervical cancer	40^±^	2.4
The Pap test screens for cervical cancer	7	3.9	I hear talk about cervical cancer on social media spaces	62^±^	2.1
There are screenings for cervical cancer	9	4.3	There were close women in my life that talked about cervical cancer	23^±^	2.5
HPV is a risk factor for cervical cancer	20	3.9	My friends approval of me getting a Pap test	21	3.7
HPV is a female health issue	22	4.3	My family would want me to get screened for cervical cancer	28	3.6
HPV can go undetected in the body for years	33	2.9	My child/children’s approval of me getting a Pap test	44	3.6
Condoms can prevent HPV	47	3.9^a^	My family would want me to get screened for cervical cancer	35	3.8
HPV stand for human papilloma virus	61	4.0	I can find health information on medical websites	58^±^	3.6
	Card #	Rating	My friends and I talk about health	52	3.8
Cervical Cancer 101 (Knowledge)		2.01	My mother talked to me about cervical cancer	26^±^	2.4 ^±^
Cervical cancer is caused by a STI	1	3.6	I know what a cervix looks like	60	2.6
Cervical cancer is caused by HPV	2	4.0	People are aware of cervical cancer	69	3.4
There is a vaccine for HPV	4	3.9	I trust my doctor	63^±^	3.2
Only females can contract cervical cancer	5	3.6^a^	I know how to find health information I need to keep myself healthy	38	3.4
The Pap test screens for HPV	15	3.0^a^	I am sure that I could go get a Pap test	51	3.8
Males can get HPV	14	3.6	I am sure that I could keep having a Pap having a Pap test, even if I had to go to another office to get one	54	3.6
Most adult females have already been exposed to HPV	24	2.2	I would like to stay up-to-date with my Pap screenings	36	4.0
Cervical cancer is preventable	29	2.6	Women’s health awareness in my community	18^±^	4.1
Males can contract cervical cancer	48 ^±^	3.6^a^	General practitioner keeps me up-to-date	25^±^	3.3
Screening will help prevent cervical cancer	64	3.9	My friend’s opinion about the choices I make is very important to me	30	3.4
Black women die from cervical cancer more than other racial groups in the U.S.	65 ^±^	2.1	I’ve heard somewhere that cervical cancer was very preventable	31^±^	2.3
	Card #	Rating		Card #	Rating
Fatalism		3.91	Ease of Prevention		2.90
If someone is meant to get a serious disease, they will get it no matter what they do	6	3.9	There are many women’s health clinics/doctor’s offices in my community	13	2.6
It is easy to get health insurance or have your medical screenings financially covered	10	3.1	Getting a Pap test is painful	41	2.5^a^
If someone has a serious disease and gets treatment for it, they will probably still die from it	11	2.3	Getting a Pap test would only make me worry	39^±^	3.5
If someone is meant to have a serious disease, they will get that disease	37	3.0	It is embarrassing to get a Pap test	43	3.0
I will die when I am fated to die	42	4.1	Getting a Pap test may be difficult for me due to my work/childcare schedules	19^±^	3.1^a^
If someone is meant to get a serious disease, it does not matter what kinds of food they eat, they will get it anyway	50	2.9	My husband’s/ spouse/ boyfriend’s/ girlfriend’s opinion about the choices I make is very important to me	34	2.7
If someone gets a serious disease, that’s the way they were meant to die	53	2.7	My mother’s opinion about the choices I make are very important to me	46	3.6
If someone was meant to have a serious disease, it does not matter what doctors and nurses tell them to do, they will get it anyway	55	3.0	Friends/colleagues who work in the health industry tell me things about my health	59^±^	3.8
How long I live is predetermined	57	3.7	Getting to doctor’s appointments is easy	27	3.4
My health is determined by something greater than myself	56	3.5	Doctor’s appointments are stressful for me, in general	32	3.0
Prayer and faith can save me no matter what the doctor says	66^±^	3.8	Health insurance would make it easier	45	4.0
			Keeping doctor’s appointments is easy	49	2.6

±, Concept/Phrase was generated by participants during Phase 1 of the concept mapping procedure; a, Item reverse coded for overarching concept rating.

Phases 2 and 3 of the concept mapping procedure consisted of each individual sorting an individual stack of card statements (or concepts) into *k* number of piles. For the sorting phase (phase 2), a single phrase appears on each numbered card. Phrases generated by phase 1 were mixed in with predetermined phrases that capture fatalism, subjective norms, self-efficacy, skills, resources, and behavior (27) related to cervical cancer and prevention. Each statement was printed individually on an index card in third-person statements (25) (e.g., “Many people have heard of cervical cancer;” “Pap test is embarrassing”). Participants were asked to sort cards in piles in a way that makes sense to them with the following rules: there is no limit to the number of piles you create, as long as there is more than one pile, and there must be more than one card per pile. After creating their piles, the participants began the rating phase (phase 3).

During the rating phase, the participants were given a rating sheet. The rating sheet allowed them to name their individual piles, record which phrases belonged together (using the number that appeared on each card) and rate the agreement (or importance, where appropriate) of each card phrase using a scale of 1—Strongly Disagree to 5—Strongly Agree using the rating sheet. When completed, the research team member collected the cards and rating sheets. Phases 2 and 3 took approximately 40 min. Participants were compensated with a $30 department store gift card upon completion of the concept mapping activity.

### Measures

Several pre-determined cards were created based on previous research (26–28), in addition to generated statements. Previously developed items were adopted from previous research and validated measures on cervical cancer knowledge and HPV knowledge (28, 29); as well as predetermination (30), self-efficacy and subjective norms toward cervical cancer prevention (27, 30, 31). The predetermined statements reflect common psychosocial constructs that have been shown to impact prevention behavior. A total of 50 pre-constructed cards reflecting psychosocial constructs were used.

During the information generating phase, phase 1, through open discussion, the participants came up with 19 additional phrases reflecting their sociocultural concepts about cervical cancer prevention. Thus, a total of 69 cards with phrases were provided for the concept mapping activity. Table 1 shows the phrases that were pre-constructed and the phrases that were generated through phase 1 inquiry. Participants were given a rating sheet with boxes to identify and name each of their piles. The rating sheet also contained a list of all card phrases and a 5-point Likert-like scale to rate the level of agreement (or importance, where appropriate) of each phrase.

### Analytic procedure

Phases 4–6 of the concept mapping procedure consists of the data analyses. A binary similarity matrix of all sorting piles and a group similarity matrix structured the conceptual domains (phase 4). Concept mapping uses multidimensional scaling to create point and cluster maps (phase 5). Similar to thematic analysis, the research team convened to discuss the optimal number of clusters by examining the different cluster solutions. The research team examined the cluster solutions [starting at 10 clusters (32)] and collectively decided on which configuration makes the most sensible clusters. Finally, the average ratings across participants for each statement in each cluster are graphically overlain onto the cluster map to yield a final concept map (phase 6). A list was then created to identify the statements that fell within a particular concept. We also discussed and named the concepts based on the statements identified within. Items were averaged on a 5-point Likert-like scale, therefore average ratings closer to 5 indicates that the women believed that particular overarching concept or individual concept was *very important* or *strongly agreed* with it; and average ratings closer to 1 indicates that the women believed the concept to be *not very important* or *strongly disagreed* with it. The concept mapping data was analyzed using Global Concept Systems, an academic and professional concept mapping software specifically designed to analyze the concept mapping procedure.

It is worth mentioning here how to read the cluster map. The colored geometrical shapes on the map represent the different concepts that emerged from the data. The more layers on the shape, the greater the *importance* of the overarching concept. The statements/phrases that the participants sorted and rated comprise the overarching concept (i.e., the geometrical shape). The larger and more spread out the shape is, the more spread out the individual items are in the minds of the participants. The smaller and more compact a shape is, the more tightly knit the concepts are. The relative positions of the overarching concepts/shapes show how related they are to one another. Lastly, the nodes (i.e., numbered dots) on each concept/shape are the individual phrases/statements. The closer the nodes are to one another, the closer the phrases/statements are in the minds of the participants.

## Results

**Figure 1:**
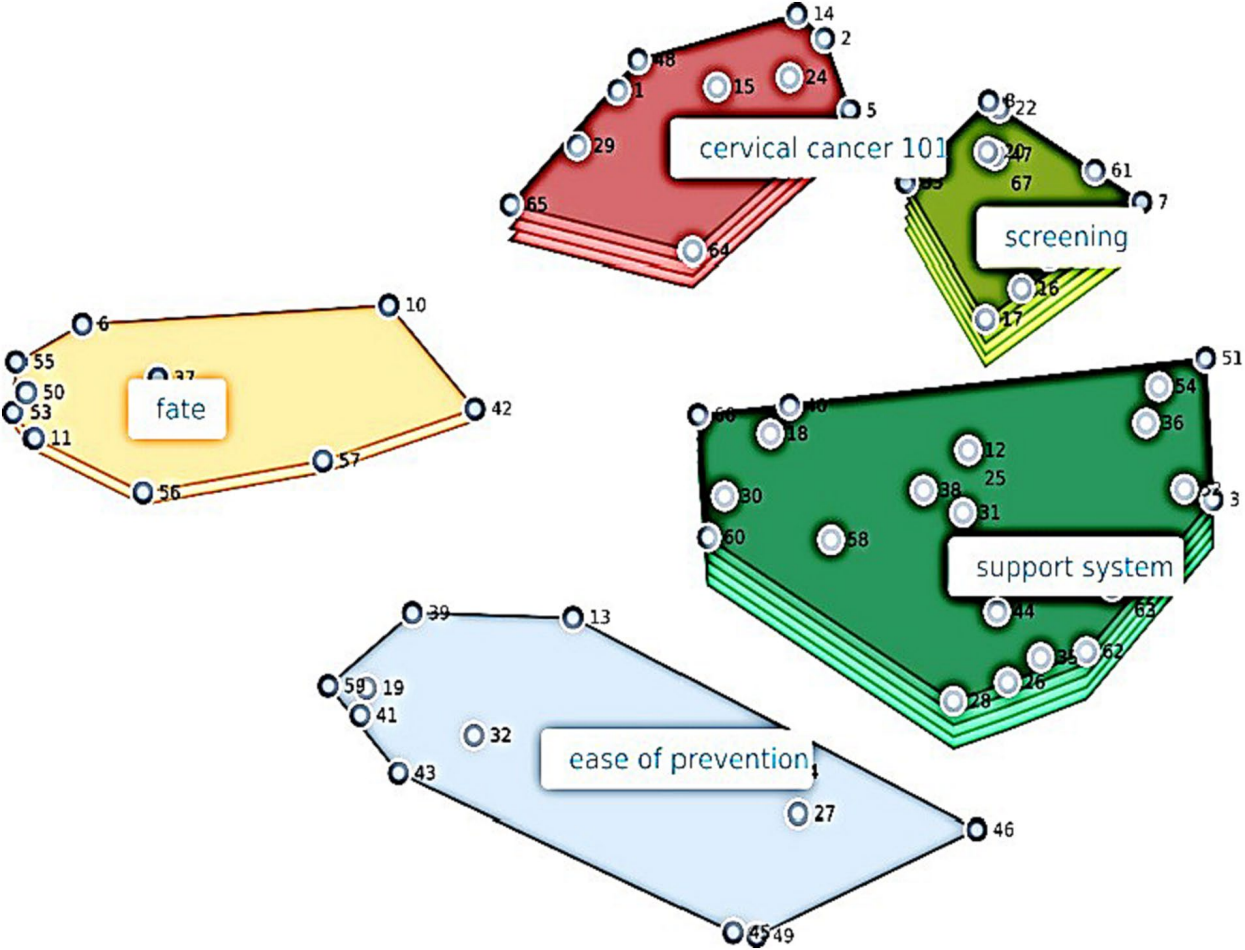
Concept mapping resulting cluster map.

After examining different cluster solutions, the research team determined that the 5-cluster cluster solution was preferred. In naming the clusters, the following cluster names were decided upon: Screening, Support System, Cervical Cancer 101 (knowledge), Fatalism, and Ease of Prevention. Figure 1, the resulting cluster map, shows how the participants, young adult African American women, conceptualized psychosocial constructs and sociocultural determinants of cervical cancer prevention behavior.

Thus, a hierarchy of importance of the overarching concepts can be observed in Figure 1: (1) and (2) Screening and Support System (same level of importance), (3) Cervical Cancer 101 (knowledge), (4) Fatalism, and (5) Ease of Prevention. Table 1 shows the overarching concepts, the individual card phrases that loaded within each, card numbers that coincide with nodes in Figure 1, rating average of the overarching concept, and average rating of the individual phrase.

We observed an average Screening rating of 3.64, which was observed as one of the most important concepts (Figure 1; 5 layers on the Screening concept). Although this concept was one of the most important, the average screening rating indicated that the women were moderately familiar with these screening concepts, but understood that these concepts were important. Observing the average individual concept ratings, the most important individual concepts within the Screening construct were: *There are screenings for cervical cancer* [4.3]; *HPV is a female health issue* [4.3]; and *Screening is important only if cancer runs in your family* [4.2]. The latter item shows that the women agreed with a false notion that screening was only important cancer runs in the family (and was reverse coded to calculate the overall Screening concept rating).

We observed an average Support System rating of 3.59, which was the other most important concept (Figure 1; 5 layers). Beliefs about their support system regarding women’s health and cervical cancer prevention were mixed. Individual concepts that participants agreed with regarding their support system were: *Health insurance would make cervical cancer screening easier* [4.1] and *Importance of women’s health awareness in my community* [4.1]; while the lowest rated individual concepts were: *I hear talk about cervical cancer on social media spaces* [2.1] and *I’ve heard somewhere that cervical cancer was very preventable* [2.3].

We observed an average Cervical Cancer 101 Knowledge rating of 2.6, which was the second most important concept (Figure 1; 4 layers). This indicated low cervical cancer knowledge in some of the individual concepts. Individual concepts that were rated highest were: *Cervical cancer is caused by HPV* [4.0]; *There is a vaccine for HPV* [3.9]; and *Screening will help prevent cervical cancer* [3.9]. The lowest rated knowledge items were *Black women die from cervical cancer more than other racial groups in the U.S.* [2.1] and *Most adult females have already been exposed to HPV* [2.1].

We observed an average Fatalism rating of 3.9, which indicated moderately fatalistic attitudes about health. The overarching Fatalism concept was the third most important concept (Figure 1; 2 layers). The highest endorsed individual fatalistic concepts were *I will die when I am fated to die* [4.1] and *If someone is meant to get a serious disease, they will get it no matter what they do* [3.9]; however, the least endorsed individual fatalistic concept was *If someone has a serious disease and gets treatment for it, they will probably still die from it* [2.3].

We observed an average Ease of Prevention rating of 2.9, which indicated that the women believed that cervical prevention is neither easy nor hard. Ease of Prevention was the least important overarching concept (Figure 1; 1 layer). However, the highest rated individual Ease of Prevention concepts were that *Health insurance would make it easier* [4.1] and *Getting a Pap test is painful* [2.2] (meaning they did not generally think so; item was reversed coded for overall Ease of Prevention concept rating), while the least agreed with individual concepts were, *Getting a Pap test would only make me worry* [2.5] and *Keeping doctor’s appointments is easy* [2.6].

## Discussion

The present study utilized a concept mapping procedure to examine the psychosocial, sociocultural, and knowledge beliefs of AAW in Houston, Texas, an area of public health interest regarding cervical cancer mortality among AAW. The procedure resulted in a 5-solution model, indicating five major areas of interest in the minds of these young adult AAW. Screening, Support System, Cervical Cancer 101 (knowledge), Fatalism, and Ease of Prevention emerged as the overarching concepts that may be addressed in cultivating a culture of health among AAW in the community.

Individual concept items that were generated by the participants in phase 1 of the concept mapping procedure mostly reflect their sociocultural needs and concerns (see Table 1 footnote for participant-generated phrases.) However, it is interesting that these sociocultural aspects of cervical cancer prevention did not load onto their own overarching concept in any of the cluster model determinations we examined. Rather, the sociocultural concepts were woven throughout the overarching concepts. (Where appropriate in the following sections, the concept rating is referenced in the brackets that follow the concept.)

Within the Screening concept, sociocultural concepts centered around cervical cancer being an issue for AAW, in particular, and the genetic aspect of cancer running in the family (33). Screening may be improved among AAW with instilling the common knowledge that cervical cancer screening plays an important role African American health, as African American women do not contract cervical cancer at disproportionate rates, but they die from cervical cancer at disproportionate rates. Public health has emphasized the importance of screening for various infectious diseases, however, more women’s health messaging on the importance of screening for women’s health concerns (cervical cancer, breast cancer, etc.) may be needed for tailored implementation in African American communities. Participants moderately agreed that *males can get HPV* [3.6]. However, we would want it to be common knowledge that HPV is the most prevalent sexually transmitted infection in the world (4). Most men who are sexually active have already acquired HPV at some point.

The Support System construct included the most sociocultural aspects of cervical cancer. The items encompassed themes of environmental support (e.g., *There are commercials about cervical cancer* [2.4] and *I hear talk about cervical cancer on social media spaces* [2.1]) as well as interpersonal support (e.g., *My mother talked to me about cervical cancer* [2.4] and *There were close women in my life that talked about cervical cancer* [2.5]). The lower ratings on these individual concepts reveals that the women do not generally believe that they have these social supports. This suggests that HPV and the associated risk of developing cervical cancer is not common knowledge in the social or family environment for most women. It is interesting that, according to the participants, knowing what a cervix looks like was an individual concept that loaded under the social support concept in all of our resulting models. More interestingly, this individual concept, *I know what a cervix looks like*, was rated low [2.6], meaning that young AAW did not know what a cervix looks like. The desire to stay up to date with Pap test guidelines also loaded under the Support System concept. This individual concept was also high with the women overall agreeing with the concept statement [4.0]. However, Figure 1 of the overall concept map shows that this overarching concept was hierarchically one of the most important overarching concepts to the participants.

Prevention knowledge is fundamental and essential to understanding how our behaviors and environments contribute to our health and well-being. Overall, the women reported a mid-range relative importance of cervical cancer knowledge and it was the second-most important concept. Yet, participants exhibited relatively low cervical cancer knowledge [overarching Cervical Cancer 101 (knowledge) rating—2.01]. Although the women were confident screening will help prevent cervical cancer and that cervical cancer is caused by HPV, they were less confident that HPV, which causes cervical cancer, is a sexually transmitted infection. Additionally, condoms are not as efficacious for the prevention of HPV particularly because HPV can be contracted through skin-to-skin contact. They were not aware that AAW die from cervical cancer more than other racial groups in the U.S., which was an individual sociocultural concept generated in phase 1. AAW are 75% more likely to die from cervical cancer, despite it being very easy to prevent when detected through having up-to-date Pap tests. Staying up to date on Pap tests will catch abnormal cells quickly and they can be removed, usually preventing the cancer from forming at a 94% prevention rate, making cervical cancer one of the most preventable human cancers (4). There was also a lack of knowledge about the transmission of HPV and contracting cervical cancer. They were not aware that most adult females were already exposed to HPV and that males cannot contract cervical cancer because the cervix is a female-only reproductive organ; although males are carriers of HPV, as the lifetime prevalence of HPV in men has been estimated at 49% for all types of HPV and 35% for high-risk strains of HPV (34).

We observed lower ratings of agreement regarding their general practitioner keeping them up to date on their recommended screenings (Social Support concept) and cervical cancer being discussed in school health (Screening concept). In conjunction with the lack of general knowledge regarding cervical cancer, this may support the observation that HPV and cervical cancer risk are largely not included as a standard topic of women’s health. Sexual health education provided in the Texas school systems may not be thoroughly comprehensive, and targeted public health information is not reaching this demographic. Thus, young AAW may be inadvertently at risk due to lack of information or misinformation regarding HPV.

Fatalistic perspectives on health have been associated with lack of self-efficacy regarding health promoting behaviors such as screening (35). Fatalism speaks to the control one feels over their life and health. Fatalism was one of the least important concepts in the minds of these young AAW, which could be interpreted as a positive finding. Also, in examining Figure 1, the Fatalism concept was farther removed from the other concepts in the minds of the participants. It is good that young AAW do not view fatalistic attitudes as very important in their conceptualization of cervical cancer prevention. However, this must be juxtaposed by their relatively high overall fatalism rating, which was 3.9, meaning that they endorsed many of the fatalistic concepts. The relatively high ratings include the notion that if someone is meant to get a serious disease, they will get it no matter what they do and that their health and lives are predetermined. Public health strives to break these beliefs as they circumvent motivation to control one’s destiny. One sociocultural concept emerged which was that God can save them despite a doctor’s grim diagnosis. While spirituality and religion can and should be positively incorporated into prevention and treatment for those to whom it benefits, this is not without understanding prevention science and adhering to treatments. Adhering to a doctor’s concerns and treatments can be viewed as tools provided by God for spiritual individuals who may succumb to fatalistic perspectives on health. This is particularly important for health psychologists, therapists, public health professionals, and other health counseling professionals, as spirituality has been shown to be a protective factor in health outcomes (35). Furthermore, previous research conducted in African American churches has shown positive outcomes for cervical and breast cancer prevention (27).

In order to obtain screening, many other steps must proceed. A woman must have the efficacy and knowledge to make an appointment, and then the skills and resources to complete the appointment, such as having transportation to arrive at the appointment and the means to pay via insurance or out-of-pocket costs. These are the kinds of concepts that encompassed the Ease of Prevention concept, which was the least important concept in the minds of the participants (Figure 1; 1 layer), relative to the other concepts. However, they did not seem to believe that cervical cancer prevention was “easy,” observed by the overarching concept rating of 2.9. Sociocultural concepts related to the ease of prevention included personally knowing people in the health industry and factoring in work schedules and childcare into scheduling a screening appointment. It is interesting that having friends and colleagues in the healthcare industry did not load under the Social Support concept, but rather that Ease of Prevention concept.

Most of the individual concepts included the logistics of actually completing a screening appointment (including having health insurance), but some subjective norm concepts such as the importance of their significant other’s and mother’s opinion about their health also loaded as a part of how young AAW conceptualize the ease of prevention. The approval of significant others and family members may affect an individual’s motivation to seek preventative care. Young women surrounded by family who regard preventative care highly may be more likely to follow through with appointments and screenings. Some cultures, including traditional African American culture, value matriarchal knowledge similarly to that of public health authorities. The cultural validity of the prevention information is important to prevention reach and increasing prevention behavior.

Although group concept mapping is sparse in community health research, particularly regarding cervical cancer prevention, these results nevertheless echo previous studies that found that understanding the opinions and beliefs of minority populations using concept mapping is fundamental to intervention and public health message development if we are to reach diverse populations (23, 25). The present exploratory study confirms and adds to similar concept mapping studies that concluded that taking into account existing social and community networks will yield greater impacts on prevention for hard-to-reach populations (23, 25). For example, our findings suggest that we are in need of more public health messaging about screening and up to date guidelines on social media sites (advertisements, social media groups, etc.) and television/radio within predominantly African American female media environments (e.g., Facebook, specific television time slots, billboards in African American communities). Concept mapping has also found to be useful for rapid implementation efforts in cervical cancer prevention (36). Along with accurate knowledge, encouraging discussions among AAW about women’s health will help facilitate a culture of health within families and the community.

### Public health and practice implications

Spreading awareness about the cervical cancer risks posed by untreated HPV infections, understanding how HPV is contracted and transmitted, and publicizing the efficacy of preventing its progression to cervical cancer is necessary to increase screening behavior. Further, improved awareness in African American communities may result in more young AAW and girls taking preventative measures such as vaccination and screening tests. Given the high rates of exposure to HPV and the high efficacy of preventative measures, young women, especially within low resource communities, need access to care. An African American woman wanting to take advantage of prevention services may not be able to do so due to many access barriers that were conceptualized in the present study. Not being able to keep a doctor’s appointment due to transportation, work schedule, and other socioeconomic factors weaken public health prevention messages. There must be a shift in public health efforts to examine the environment and how we can create a culture of health for AAW that indeed encompasses access to care and a deep consideration for cultural and socio-environmental barriers.

Having access to this information prior to becoming sexually active provides the best chance of creating a culture of health and preventing HPV from developing into cervical cancer. Creating a more comprehensive standard for sexual health education in Texas schools would raise awareness for HPV and cervical cancer. Including women’s health as a part of school health education would likely increase the chances that young women will seek preventative care (e.g., HPV vaccine) before becoming sexually active. Offering HPV vaccinations to youth in schools would assist in a culture of health, as it may boost collective efficacy among young girls, and possibly allow them to feel more comfortable in a familiar setting such as school. Tailored advertisements on social media platforms may also be a more accessible way for young women to learn about HPV and the associated risk of cervical cancer. Therefore, social media environments should also be explored as an avenue to increase cervical cancer prevention. Public health and community support for women’s health is also needed in African American communities. Although we favor free/low costs screening for all females within the prevention guidelines, offering free HPV vaccinations, HPV tests, and/or Pap tests for uninsured women via mobile clinics, for example, would strengthen women’s health equity in these communities. Overall, properly disseminating preventative literature to this population using culturally tailored approaches, incorporating cultural strengths, and addressing sociocultural strengths and barriers are essential in increasing cervical cancer screening behaviors among AAW.

### Limitations and future research

The limitations of the present study include a low sample size and alternate interpretations of the data. While there is no required sample size for the group concept mapping procedure, our sample of 15 participants cannot be generalized and should be taken with caution when applying to other populations with varying demographic characteristics than the sample use for the present study. The present study serves as an exploratory method of inquiry that may be used for prevention program development. Although there was no rationale to restrict participation to the 18-25 age range, it may be a function of the spaces where recruitment was carried out (health fairs held in the park, word-of-mouth, and on social media sites). As the present study is exploratory, future research could determine whether different results would be garnered from varying ages of African American women. Recruitment in spaces with a wider typical age range may be beneficial also. Group-based concept mapping is inherently a mixed-methodology; meaning that the data configures to the specified number of overarching concepts requested, and it is up to the research team to decide the most appropriate configuration that “makes sense.” Thus, there are hypothetically other interpretations that could have been made.

Social and cultural factors influence how we think about our health and likewise how we conceptualize its importance. Future research should further address how cervical cancer prevention is impacted by sociocultural factors and how research can capitalize upon the social environment. Even when information is available, but is not culturally competent/appropriate/appealing, the prevention message may not “reach” the intended audience nor be as effective. More research is needed to corroborate these findings. Nonetheless, the present findings may be used to generate hypotheses and/or enhance prevention presence in the community through effective messaging for cervical cancer among young African American women.

## Data availability statement

The datasets presented in this article are not readily available because the present study was conducted as an exploratory feasibility study and used a very small sample size (though adequate for the methodology). To protect the confidentiality and anonymity of participants, data sharing is not allowable. Requests to access the datasets should be directed to ccarmack@central.uh.edu.

## Ethics statement

The studies involving humans were approved by the University of Houston Institutional Review Board. The studies were conducted in accordance with the local legislation and institutional requirements. The participants provided their written informed consent to participate in this study.

## Author contributions

CC: Formal analysis, Funding acquisition, Investigation, Methodology, Software, Supervision, Visualization, Writing – original draft, Writing – review & editing. AR: Data curation, Supervision, Validation, Visualization, Writing – review & editing. TC: Project administration, Supervision, Writing – review & editing. SM: Writing – review & editing.
